# The bioaccessibility of eicosapentaenoic acid was higher from phospholipid food products than from mono- and triacylglycerol food products in a dynamic gastrointestinal model

**DOI:** 10.1002/fsn3.58

**Published:** 2013-09-05

**Authors:** Nobuhiko Domoto, Marjorie E Koenen, Robert Havenaar, Akihiro Mikajiri, Boon-Seang Chu

**Affiliations:** 1 Nippon Suisan Kaisha, Tokyo Innovation Center, Nanakuni HachiojiHachioji, Tokyo, Japan; 2 Nippon Suisan Europe, Ouderherk aan de AmstelZeist, The Netherlands; 3 Netherlands Organization for Applied Scientific Research (TNO)Zeist, The Netherlands; 4 Nippon Suisan Kaisha, Fine Chemicals General PlantTokyo, Japan; 5 Institute of Bioproduct Development, Universiti Teknologi MalaysiaSkudai, Malaysia

**Keywords:** Bioaccessibility, eicosapentaenoic acid, in vitro digestion, monoacylglycerol, phospholipid, PUFA, triacylglycerol

## Abstract

The bioaccessibility of eicosapentaenoic acid (EPA) in the forms of monoacylglycerol (EPA-MAG), triacylglycerol (EPA-TAG), and phospholipid (EPA-PL) during gastrointestinal passage was compared in this study using a dynamic gastrointestinal model (TIM system). The TIM system simulated the average upper gastrointestinal tract conditions of healthy human adults after intake of a meal (fed state conditions). In this study, the three EPA-rich oils were separately homogenized with full fat milk to obtain oil-in-water emulsions. Plain yogurt was added into the mixture at an emulsion/yogurt ratio of 4:1 (w/w) as the food matrix of the test products. The results show that the test meals containing EPA-PL left the stomach compartment most efficiently in comparison with the gastric emptying of EPA-MAG and EPA-TAG. The PLs also showed a significantly (*P* < 0.05) higher bioaccessibility of EPA (75–80%) in comparison with MAG (30%) and TAG (38%). The better gastric emptying of EPA-PL was likely related to the more stable emulsion of EPA-PL in the test meal. EPA-PL was delivered within the meal matrix into the duodenum instead of floating on the top of the test meal matrix. EPA-MAG had the highest amount of EPA that did not leave the stomach (68% of the test meal). The results from this work indicate that EPA-PL is a more effective form of EPA for a higher lipid bioaccessibility than MAG and TAG under the test conditions.

## Introduction

Omega-3 long-chain polyunsaturated fatty acids (PUFA), particularly eicosapentaenoic acid (EPA) and docosahexaenoic acid (DHA), are important building blocks of phospholipid (PL) membranes in tissues throughout the body (Connor 2000). This is especially true in the retina, brain, and spermatozoa, in which DHA constitutes up to 36% of total fatty acids of the cell membrane (Neuringer et al. 1986; Lin et al. 1993). There is also evidence of inverse relationships between DHA and EPA in the diet with the occurrence of coronary heart disease (Din et al. 2004; Breslow 2006; Gebauer et al. 2006). Meta-analyses from randomized clinical trials have concluded that the consumption of EPA and DHA at doses >3 g/day can improve cardiovascular disease risk factors, including decreasing plasma triacylglycerol (TAG) concentrations, blood pressure, platelet aggregation, as well as improving vascular reactivity (Breslow 2006). DHA and EPA have also been associated with reduced risk of inflammatory and autoimmune diseases like atherosclerosis, rheumatoid arthritis, asthma, and Alzheimer's disease (Connor 2000; Calder 2006; Cleland et al. 2006). However, considerably high amounts of fish would have to be consumed to achieve adequate intake of EPA and DHA for their therapeutic benefits (Wakil et al. 2010).

A good strategy to raise plasma concentrations of the PUFA is by supplementation of fish oil containing high concentrations of EPA and DHA in the form of soft gels or emulsions (Haug et al. 2011). Other approaches to improve the digestibility and absorption of EPA and DHA include lipid structure reformulation. Bioavailability of EPA and DHA is dependent on the intramolecular triacyglycerol structure and lymphatic transport. Total lymph lipids of EPA and DHA are significantly higher if the PUFA are esterified at the *sn*-2 position of the TAG compared to those at *sn-*1 or *sn*-3 positions (Christensen et al. 1995). Wakil et al. (2010) have demonstrated that transesterified fish oil experiences higher EPA/DHA bioavailability compared to the natural nontransesterified fish oil. It is speculated that mono- and diacylglycerols, the coproducts of transesterification, facilitate intestinal digestion and act as emulsifying agents, which in turn promote the uptake of EPA and DHA (Wakil et al. 2010). These results show the important influence of different molecular structures of EPA and DHA lipid sources on their digestion and absorption.

This study compares the in vitro bioaccessibility of EPA in the form of acylglycerols and PL in a yogurt food matrix. Marine PL such as krill oil is potentially a good dietary source of EPA and DHA. The fatty acids in PL are mostly bound to the choline head-group, in contrast to fish oil in which the fatty acids are esterified to the glycerol backbone (TAG). The PLs are polar lipids and form α-lamellar bilayer structures when hydrated, in contrast to TAGs which are not surface-active. Therefore, even if the fatty acid composition of the two lipids is similar, their chemical nature is very different. It is of interest to investigate how the chemical structure and interfacial behavior of the lipids influence the lipid digestion and in turn affect the bioaccessibility of EPA and DHA.

In this study, fish oil and PL-PUFA were prepared as oil-in-water emulsions. The main objective of the work was to compare the bioaccessibility of EPA in the form of monoacylglycerol (MAG), TAG, and PL during transit through a dynamic gastrointestinal model (TIM system, Minekus et al. 1995). The results will provide a better understanding on the comparative in vitro digestibility of the lipids and bioaccessibility of EPA.

## Material and Methods

### Materials

Three EPA-rich oils, in the forms of MAG (36% purity, major remaining components were diacylglycerols and TAGs; peroxide value, PV = 1.23 mEq/kg), TAG (100% purity, PV = 0 mEq/kg), and PL (95% purity, PV = 2.30 mEq/kg) were obtained from Nippon Suisan Kaisha, Ltd. (Tokyo, Japan). Full fat milk (3.5% fat; FrieslandCampina, Amersfoort, The Netherlands) and plain yogurt (2.9% fat; FrieslandCampina) were purchased from a local supermarket. Heptadecanoic acid and methanolic boron trifluoride for gas chromatographic determination of fatty acids were purchased from Merck (Merck-Millipore, Schiphol-Rijk, The Netherlands). All other chemicals used were of analytical grade purchased from Sigma-Aldrich (Saint Quentin Fallavier, France).

### Test meal preparation

The test meals were prepared by vigorously mixing, under nitrogen flushing, a full fat milk with EPA-rich oil test products using an ultra turrax mixer (model CAT X620; Zipperer GmbH, Staufen, Germany) for 5 min at 11,000 rpm and squeezed through a thin tube to get small fat droplets. Plain yogurt was added to the mixture at a milk/yogurt ratio of 4:1 (w/w) and homogenized with a spoon. Four test meals were prepared which, respectively, contained 0.2% w/w EPA-MAG, 0.2% w/w EPA-TAG, 0.2% w/w EPA-PL-0.2, and 0.3% w/w EPA-PL-0.3. The fatty acid compositions of MAG, TAG, and PL are shown in Table 1. These test meals were prepared less than 1 h before the experiment. Prior to use, the meal was homogenized in simulated oral fluid (5:1 w/w) prepared by aliquoting 6.2 g/L sodium chloride, 2.2 g/L potassium chloride, and 0.3 g/L calcium chloride dihydrate. A total of 300 g of this homogenized product (250 g test meal + 50 g simulated oral fluid) was transferred into the gastric compartment of the dynamic gastrointestinal model.

**Table 1:** Fatty acid composition (mol%) of the eicosapentaenoic acid (EPA)-rich oils in the forms of monoacylglycerol (MAG), triacylglycerol (TAG), and phospholipid (PL)

Fatty acids	MAG (mol%)	TAG (mol%)	PL (mol%)
C14:0	2.02	5.25	2.87
C15:0	0.00	0.28	0.23
C16:0	3.09	6.60	26.66
C16:1 (n-7)	2.40	8.41	2.66
C18:0	1.12	0.59	1.70
C18:1 (n-9)	5.26	7.91	12.18
C18:2 (n-6)	0.33	1.13	1.24
C18:3 (n-3)	0.00	1.11	0.74
C18:4 (n-3)	0.67	4.85	1.72
C20:1 (n-9)	1.43	0.77	0.85
C20:4 (n-6)	3.27	1.51	0.51
C20:5 (n-3)	42.87	28.80	31.34
C22:1 (n-9)	1.95	0.62	2.02
C22:5 (n-3)	5.83	2.92	0.68
C22:6 (n-3)	21.59	12.51	12.31

Under in vivo conditions, the differences in composition of the meals may slightly affect the postprandial conditions in the gastrointestinal tract. In the TIM system (described in the following section), we used the same computer protocol for optimal comparison of the results.

### Test system: dynamic gastrointestinal model (TIM system)

**Figure 1:**
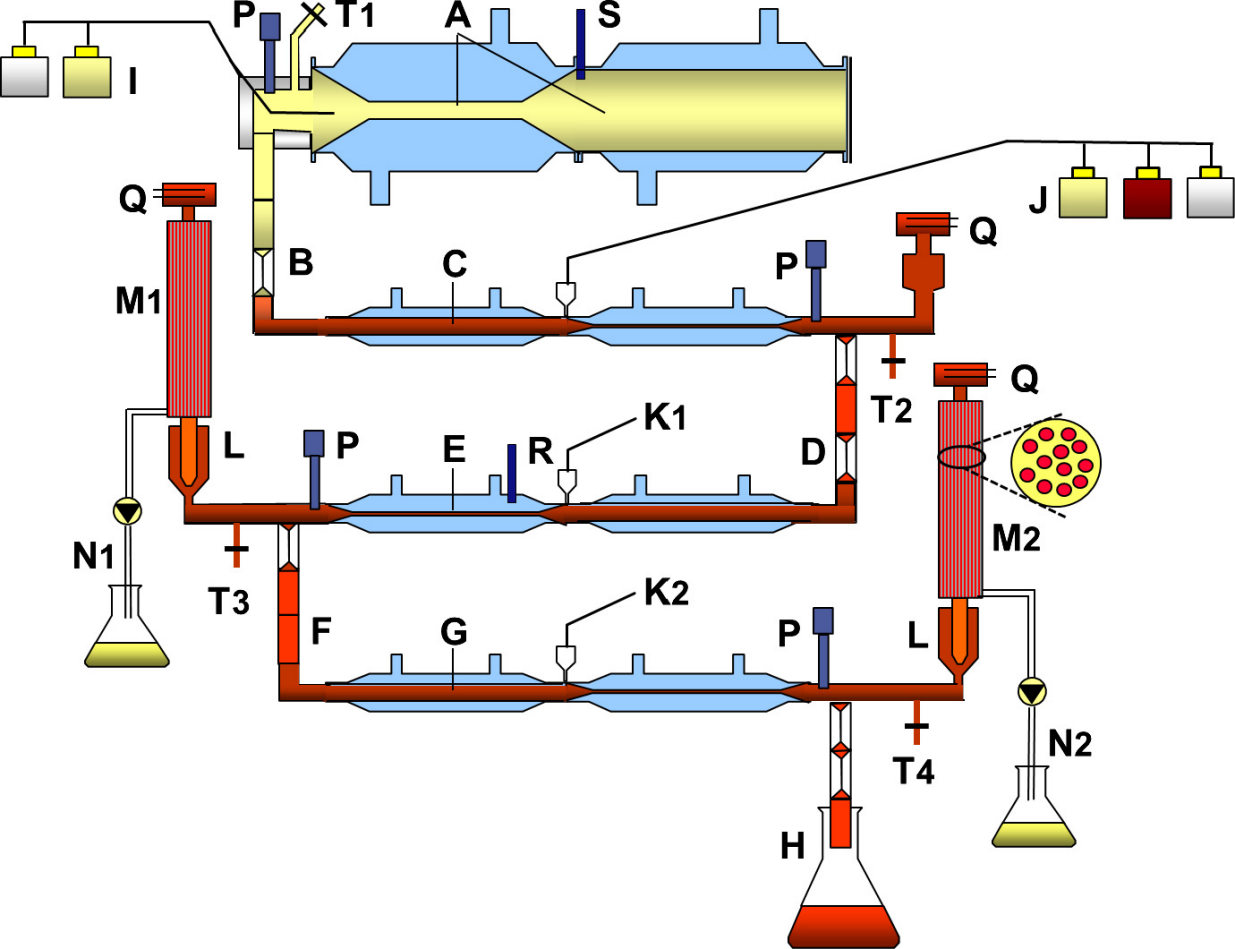
TIM system, simulating the upper gastrointestinal tract for fat digestion studies: A, stomach compartment; B, pyloric sphincter; C, duodenum compartment; D, peristaltic valve; E, jejunum compartment; F, peristaltic valve; G, ileum compartment; H, ileocecal sphincter with ileum effluent; I, stomach secretion; J, duodenum secretion; K1 and K2, jejunum and ileum secretion, resp.; L, prefilter; M1 and M2, semi-permeable membrane; N1 and N2, collection of filtration fluid from jejunum and ileum, respectively; P, pH electrodes; Q, level sensors; R, temperature sensor; S, pressure sensor; T1, gastric intake and sampling port; T2–T4, duodenum, jejunum, and ileum lumen sampling ports, respectively.

**Figure 2:**
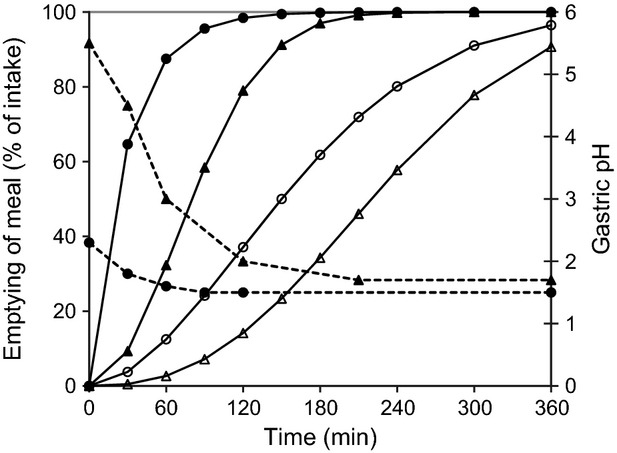
Typical average physiological gastric emptying curves (solid lines, closed markers), ileum effluent curves (solid lines, open markers), and gastric pH curves (dotted lines) for human adults in the fasted state (•○) and fed state (▲Δ) after the intake of a high fat meal as used in this study.

The study was performed in the dynamic, multicompartmental, computer-controlled system of the stomach and small intestine (TIM system TNO, Zeist, The Netherlands) as schematically presented in Figure 1 and described by Minekus et al. (1995) and applied in previous lipid and cholesterol digestion studies (Minekus et al. 2005; Reis et al. 2008). The model simulated very closely the successive dynamic conditions in the gastrointestinal tract, such as body temperature, pH, concentrations of electrolytes, activity of enzymes in the stomach and small intestine, concentrations of bile salts from fresh pig bile (for the production of micelles), and the peristaltic kinetics of mixing and transit of the contents through the gastrointestinal tract as described in more detail by Reis et al. (2008). Specific filtration systems (MiniKros M80S-300-01P module; Spectrum Laboratories Europe, Breda, The Netherlands) with a cut-off of 0.05 μm and surface area of 3100 cm^2^ were connected to the jejunum and ileum compartments of the model. Via these membranes products of lipid digestion and lipophilic compounds that were incorporated in micelles were removed to determine the bioaccessible fraction of fatty acids. The experiments in the TIM system were performed simulating the average physiological conditions in the upper gastrointestinal tract of healthy human adults after the intake of a meal (fed state conditions). These conditions include especially the dynamics of peristaltic mixing, gastric emptying and intestinal transit times, gastric and intestinal pH values (Fig. 2), as well as the composition and activity of the secretion products.

### Digestibility and absorption studies

For each experiment, the secretion products (e.g., gastric juice with enzymes, electrolytes, dialysis liquids, bile, pancreatin) were freshly prepared, the pH electrodes were calibrated, and new filtration membranes (hollow fiber units) were hooked-up. The experiments were performed with continuous flushing with 100% nitrogen gas in order to avoid oxidation of EPA. Directly after filling the gastric compartment (Fig. 1, port T1) with the test meal the experiment started. The gastric content was gradually delivered into the small intestine via the “pyloric sphincter” over a period of 3 h (Fig. 1, part B). After 6 h, ∼80–90% of the small-intestinal content was gradually delivered into the “large intestine” (sampling bottle with ileum effluent) via the “ileo-cecal sphincter” (Fig. 1, part H). The pH values under average conditions in the gastrointestinal tract were simulated. The gastric pH decreased from 5.5 to 1.7 in 120 min. The pH was 6.5 in the duodenum, 6.8 in the jejunum, and 7.2 in the ileum. The experiments were performed in duplicate.

### Sampling

At the start of the experiment a sample was taken from the intake port (Fig. 1, sampling port T1) and stored at −20°C until analysis. The ileum effluent was collected on ice at 2-h intervals (time points 0–2, 2–4, and 4–6 h) by changing the sampling bottle (Fig. 1, sampling port H). The volume of each sample was measured and stored. The filtrate of the jejunum and ileum compartments was collected in 1-h intervals for 6 h (Fig. 1, sampling ports N1 and N2, respectively). The total volumes were measured and duplicate samples of 25 mL were taken and stored at −20°C until analysis. At the end of the experiments the stomach and duodenum compartments and separately the jejunum and ileum compartments were rinsed with warm water and 50% ethanol to recover as much residual fat as possible. The volume of the two collected residue samples was measured and then saponified in ethanolic KOH (1:1 v/v) at 70°C for 60 min and stored at −20°C until analysis.

### Total fatty acid and EPA determination

After saponification the samples were derivatizated for gas chromatographic analysis with methanolic boron trifluoride solution (20% m/V) at 100°C for 3 min to their methyl esters. Heptadecanoic acid was used as the internal standard; it has a response factor (RF) of 1 relative to the fatty acids. The samples were analyzed in a gas chromatograph with flame ionization detector (Thermo Electron Trace GC Ultra; Interscience, Breda, The Netherlands) with a 50 m CP Sil-18 column with an inner diameter of 0.25 mm (Chrompack, Middelburg, The Netherlands) with helium at a flow rate of 1.2 mL/min. The temperature program was as follows: from 50°C for 4 min to 160°C in 11 min, from 160°C to 195°C in 23 min, and to 225°C in 6 min and then back to 50°C. The detector temperature was kept at 300°C. A standardized batch of infant nutrition, stored at −18°C in vacuum-sealed bags was used as control. The individual fatty acids in the samples were well separated in the chromatograms to determine the concentration of EPA and total fatty acids. The Empower-2 data system (Waters, Etten-Leur, The Netherlands) was used for calculation of the surface area under the curve. All samples were analyzed in duplicate with less than 3% difference between the duplicates.

### Data analysis

All fatty acids were calculated as area under the curve relative to the heptadecanoic acid internal standard. The total fatty acids were calculated as the sum of all analyzed fatty acids (C8 up to C22). For C8 and C10 the mean (*n* = 6) RF was calculated. For the other fatty acids the RF was 1.0.

The concentration of the test compound (mg/L) in the TIM samples was multiplied by the volume of the total collected sample per time period, resulting in absolute amounts (mg) per unit time. The results were expressed as percentage of the initial dose in the stomach compartment corrected for the total recovery of the test compound. Based on duplicate experiments the mean and range between the duplicate values were calculated for each sample. Bioaccessibility in these experiments is defined as the fraction of the compound that is potentially available for small-intestinal absorption. In the case of lipids, it is assumed that products in the micellar phase are available for absorption. The used membrane system allows micelles with free fatty acids to pass through, but not the undigested fat (tri- and diacylglycerols). To calculate the bioaccessibility as percentage of total recovery, the amount of EPA per time period in jejunum and ileum filtrates was divided by the analyzed total amount of EPA of in all samples multiplied with 100. The nonbioaccessible fraction was calculated as the amount of EPA in the effluent and the residues relative to the analyzed total recovery of EPA.

Bioaccessibility in the TIM system is also expressed as a percentage of duodenal effluent (%DE). DE is all material that has passed through the stomach and duodenum compartments and is delivered into the jejunum. In this way, only the material exposed to digestion and filtration in the jejunum and ileum compartments was taken into account, and not the fat in the residue sample retained in the stomach and duodenum at the end of the experiment. The digestibility of lipids and the bioaccessibility of fatty acids were determined by calculating the distribution of total fatty acids. The data for EPA and total fatty acids were statistically analyzed with the Student's *t*-test using Excel software.

## Results and Discussion

**Figure 3:**
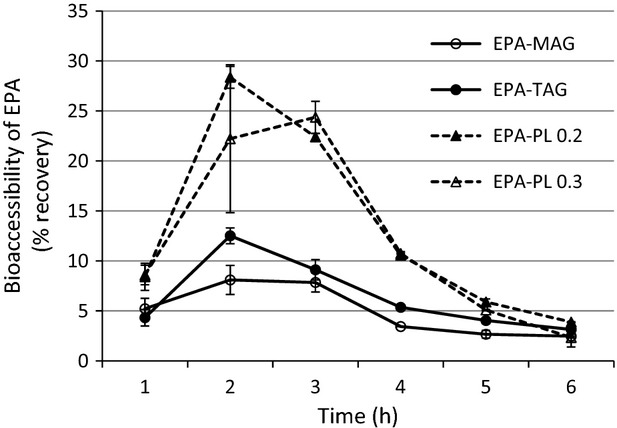
Mean (±range; *n* = 2) bioaccessibility of eicosapentaenoic acid (EPA) as percentage of recovery in the jejunum plus ileum filtrates during 6 h of digestion in the TIM system, simulating the fed state gastrointestinal conditions after the intake of a milk-yogurt meal with monoacylglycerol (EPA-MAG), triacylglycerol (EPA-TAG), phospholipids 0.2% (EPA-PL-0.2), and phospholipids 0.3% (EPA-PL-0.3).

**Figure 4:**
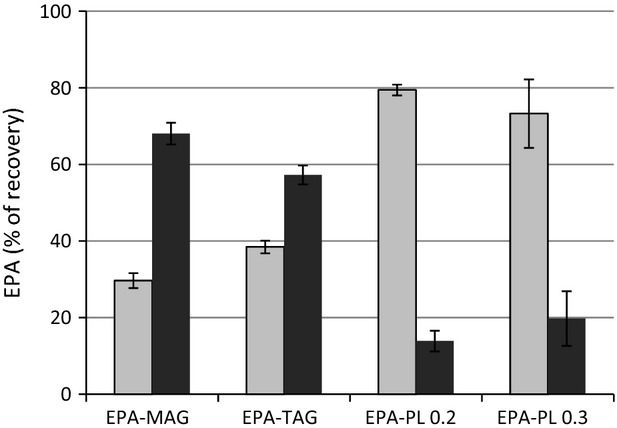
Mean (±range; *n* = 2) total bioaccessible amount of eicosapentaenoic acid (EPA) as percentage of recovery in the jejunum plus ileum filtrates (gray) and amount of EPA in gastric plus duodenal residue (black) after 6 h of digestion in the TIM system, simulating the fed state gastrointestinal conditions after the intake of a milk-yogurt meal with monoacylglycerol (EPA-MAG), triacylglycerol (EPA-TAG), phospholipids 0.2% (EPA-PL-0.2) and phospholipids 0.3% (EPA-PL-0.3).

For the four experiments performed in duplicate the mean total recovery (mass balance) for EPA was 94 ± 27% (*n* = 8). For the total fatty acids (including endogenous fatty acids from bile) the mass balance was 78 ± 11%, which is similar to the 79 ± 8% reported by Minekus et al. (2005). Due to the variability in amount of intake and total recovery of EPA and total fatty acids, the data were calculated as percentage of total recovery. The bioaccessibility of EPA as percentage of total recovery per unit of time from the four different test products in the jejunum plus ileum filtrates is shown in Figure 3. In the samples collected from 1 to 2, 2 to 3, and 3 to 4 h the bioaccessible amount of EPA from both PL products (EPA-PL-0.2 and EPA-PL-0.3) was significantly higher than that of the EPA from EPA-MAG (*P* < 0.01) and EPA-TAG (*P* ≤ 0.05). During all experiments the highest bioaccessible amount of EPA was found in the samples filtered from the jejunum compartment from 60 to 120 min (*T*_max_) and from the ileum compartment between 60 and 180 min. It was noticed that EPA-PL behaved differently compared to EPA-MAG and EPA-TAG. The amount of EPA from the PLs was higher in the filtrate collected from 0 to 6 h (*P* ≤ 0.05) and lower in the stomach and duodenum residues at the end of the experiment (*P* < 0.05) as compared with EPA from MAG and TAG (Fig. 4). The total bioaccessibility of EPA from EPA-PL-0.2 and EPA-PL-0.3 was 79 ± 2% and 73 ± 9% (n.s.) while it was 30 ± 2% and 38 ± 2% (n.s.) for EPA-MAG and EPA-TAG, respectively. On the other hand, the amount of EPA in the residues recovered from the stomach plus duodenum compartments was 14 ± 3% and 20 ± 7% EPA (n.s.) for EPA-PL-0.2 and EPA-PL-0.3, respectively, as compared to 68 ± 3% and 57 ± 3% (n.s.) for EPA-MAG and EPA-TAG, respectively (Fig. 4). It was found that the PL form of EPA was emptied more effectively from the stomach compartment into the small intestine than the acylglycerol-EPA, resulting in a much higher bioaccessibility. The study by Reis et al. (2008) also demonstrated that the amount of caprylic acid in the stomach was lower in the presence of lysophospholipids than in the control without surfactant or in the presence of monopalmitin. On the other hand, the amount of caprylic acid in the duodenum was higher in the presence of lysophospholipids in comparison with the control and monopalmitin. This indicates a higher rate of gastric emptying in the presence of PLs as found in our study for EPA-PL.

When the bioaccessibility of EPA is calculated as percentage of duodenum effluent the bioaccessibility from all four products was 90–93%. The efficient digestion and absorption from the small intestine was also demonstrated by the low amount of EPA that passed the small intestine into the colon via the ileo-cecal valve (2–5%) and the low amount of EPA in the intestinal residues at the end of the experiments (1–2%). This rapid digestion and absorption indicate that the differences in bioaccessibility of EPA between these test products were determined by differences in gastric emptying.

**Figure 5:**
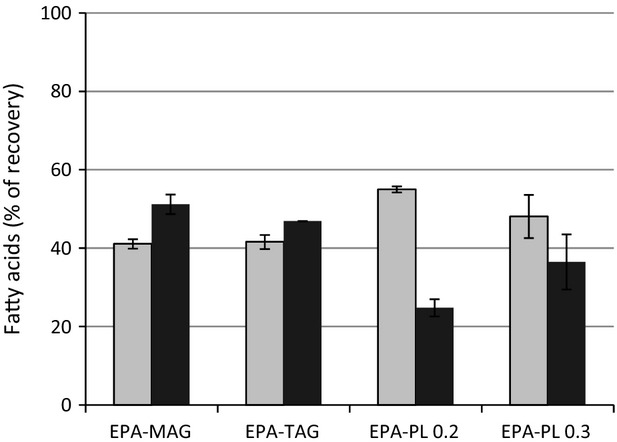
Mean (±range; *n* = 2) total bioaccessible amount of fatty acids as percentage of recovery in the jejunum plus ileum filtrates (gray) and amount or fatty acids in gastric plus duodenal residue (black) after 6 h of digestion in the TIM system, simulating the fed-state gastrointestinal conditions after the intake of a milk-yogurt meal with monoacylglycerol (EPA-MAG), triacylglycerol (EPA-TAG), phospholipids 0.2% (EPA-PL-0.2), and phospholipids 0.3% (EPA-PL-0.3). EPA, eicosapentaenoic acid.

The same observation was found for total fatty acids, but to a lesser extent (Fig. 5). The total fatty acids were also more effectively emptied from the stomach when mixed with PLs than when mixed with acylglycerols (*P* = 0.02 for EPA-PL-0.2), resulting in a slightly higher bioaccessibility of total fatty acids for the PL experiments (*P* ≤ 0.02 for EPA-PL-0.2). The difference was smaller than for EPA, due to the endogenous fatty acids from fresh bile secreted into the duodenum compartment during the experiment (∼20% of all fatty acids). The bioaccessibility of total fatty acids as percentage of duodenum effluent from the four products was 75–85%. These data are comparable with the bioaccessibility of 79 ± 8% for fat from yogurt with 3% sun flower oil and of 82 ± 3% for conjugated linoleic acid as reported by Minekus et al. (2005) and Gervais et al. (2009), respectively, in their TIM experiments. The bioaccessibility of total fatty acids was on average 13% points lower than for EPA. These findings support the data described by Gervais et al. (2009) that the bioaccessibility for (poly)unsaturated fatty acids was ∼10–12% points higher than for saturated counterpart fatty acids.

It was reviewed by Mu and Porsgaard (2005) that the gastric emptying of TAGs in animal and human studies depends on many factors, such as amount of fat, droplet size, meal matrix, and chain length and rate of saturation of the fatty acids. However, not much is known about the effects of PUFAs and specific emulsions. In a human study (Robertson et al. 2002) it was demonstrated that n-3 PUFAs (analyzed via added ^13^C octanoic acid in breath test) were gradually emptied from the stomach. The cumulative recovery of ^13^C octanoic acid in expired breath, as measure for the gastric emptying rate of n-3 fatty acids, was 12% of the dose during the first 1 h and 28% and 42% of the dose after 2 and 3 h, respectively. The cumulative intestinal bioaccessibility of EPA-PL in this TIM study was 8%, 33%, and 55% after 1, 2, and 3 h, respectively. The similarity between the human study and the TIM study for gastric emptying suggests that we reliably mimic the gastric mixing and emptying rate. The physical aspects in relation to the stability of the emulsion determine how effectively the fat and fatty acids are emptied. The better gastric emptying of EPA-PL in comparison with EPA-MAG and EPA-TAG was expected because the PLs mixed well in milk and yogurt and formed a stable emulsion, even at lower gastric pH. The fatty acids within the stable emulsified meal matrix were delivered together into the duodenum, instead of being separated from the meal matrix and floating on the top of the test meal matrix as described by Marciani et al. (2009). Therefore, the fatty acids were delivered more efficiently to the duodenum where they mixed with the bile salts. PLs (lecithin) are frequently used as food emulsifiers. This might be an explanation for the higher EPA bioaccessibility in the jejunum and ileum of the PL form of EPA.

There were substantial differences in fatty acid composition, especially the content of EPA, of the EPA-MAG, EPA-TAG, and EPA-PL tested in this study (Table 1). EPA-MAG consisted of 42.9% mol of EPA, while EPA-TAG and EPA-PL contained 28.8% mol and 31.3% mol EPA, respectively. It is difficult, if not impossible, to standardize the fatty acid composition of the oil samples without chemically modifying the oils (such as transesterification). Despite containing the highest amount of EPA among the samples, EPA-MAG suffered the lowest EPA bioaccessibility. This is interesting as the results suggest that the abundance of EPA per se in the oil does not affect much its bioaccessibility in stomach emptying, but rather the lipid structure of which the EPA chemically bound to is of more importance. The presence of other fatty acids/lipids in the oil samples, however, may influence the overall stability of the emulsion test meal and hence EPA bioaccessibility. Perhaps one of the future challenges is to quantify such influence, and to investigate the interactions of EPA with other lipid components in the oil that affect EPA bioaccessibility.

## Conclusion

The bioaccessibility of EPA in the forms of PLs and acylglycerol oils during gastrointestinal passage was compared in this study using the TIM system simulating the average upper gastrointestinal tract conditions of healthy human adults under fed state conditions. The milk/yogurt emulsion containing PL-EPA left the stomach compartment most efficiently (over 80% emptying), in contrast to the gastric emptying of the acylglyceride forms of EPA, which were emptied from the stomach for ∼40%. The PL-EPA formed a stable emulsion, which is likely due to the fact that PLs are emulsifiers. It was very likely that the better gastric emptying of EPA-PL-0.2 and EPA-PL-0.3 was related to the more stable emulsion of EPA in the meal matrix of milk and yogurt, even during decreasing gastric pH values. EPA-MAG had the highest amount of EPA that did not leave the stomach. This was probably because the emulsion was not very stable, and when gastric acid was secreted into the stomach compartment, the emulsion began to break down and the EPA started to float on top of the stomach contents. PL-EPA seems to be an excellent product for delivering EPA into the body as the emulsion is stable; emptying from the stomach with the meal is more efficient than the other forms of EPA and it has a higher bioaccessibility in the small intestine.
